# Digital Solution to Support Medication Adherence and Self-Management in Patients with Cancer (SAMSON): Pilot Randomized Controlled Trial

**DOI:** 10.2196/65302

**Published:** 2025-02-19

**Authors:** Thu Ha Dang, Nilmini Wickramasinghe, Prem Prakash Jayaraman, Kate Burbury, Marliese Alexander, Ashley Whitechurch, Mitchell Dyer, Stephen Quinn, Abdur Rahim Mohammad Forkan, Penelope Schofield

**Affiliations:** 1 Department of Psychological Sciences School of Health Sciences Swinburne University of Technology Melbourne Australia; 2 Health Services Research and Implementation Sciences Peter MacCallum Cancer Centre Melbourne Australia; 3 Digital Health Cooperative Research Centre Sydney Australia; 4 Optus Chair Digital Health La Trobe University Melbourne Australia; 5 Department Health and Bio Statistics School of Health Sciences and Iverson Health Innovation Research Institute Swinburne University of Technology Melbourne Australia; 6 Epworth Healthcare Melbourne Australia; 7 Factory of the Future and Digital Innovation Lab Department of Computer Science and Software Engineering, School Software and Electrical Engineering Swinburne University of Technology Melbourne Australia; 8 Tasmanian Health Services Department of Health Hobart Australia; 9 Digital and Healthcare Innovation Peter McCallum Cancer Centre Melbourne Australia; 10 Sir Peter MacCallum Department of Oncology The University of Melbourne Melbourne Australia; 11 Pharmacy Department Peter MacCallum Cancer Centre Melbourne Australia; 12 Department of Clinical Haematology Peter MacCallum Cancer Centre Melbourne Australia; 13 Department of Health Science and Biostatistics Swinburne University of Technology Melbourne Australia; 14 Digital Innovation Lab Department of Computer Science and Software Engineering, School Software and Electrical Engineering Swinburne University of Technology Melbourne Australia; 15 School of Computing, Engineering and Mathematical Sciences La Trobe University Melbourne Australia; 16 Department of Psychological Sciences and Iverson Health Innovation Research Institute Swinburne University of Technology Melbourne Australia

**Keywords:** home-based cancer treatment, smartphone app, oral chemotherapy, patient safety, SAMSON, mobile phone, digital solution, medication adherence, self-management, cancer, randomized controlled trial, RCT, pilot study, oncology, mobile health, mHealth, quality of life, eHealth

## Abstract

**Background:**

Medication nonadherence is a serious problem in cancer, potentially impacts patients’ health outcomes and health care costs. Although technology-based medication adherence (MA) interventions have emerged, evidence supporting their quality and effectiveness remains limited.

**Objective:**

This study tested the acceptability, feasibility, and potential effects of Safety and Adherence to Medications and Self-care Advice in Oncology (SAMSON), a digital solution designed to support MA and self-management in cancer.

**Methods:**

A 12-week, 2-arm, unblinded, pragmatic pilot randomized controlled trial was conducted. Adults with hematological malignancies who started oral cancer medicines within the last 12 months were recruited from a metropolitan specialized hospital and randomized 1:1 to SAMSON or control (usual care). The SAMSON solution included a smartphone app with tailored alerts and real-time self-care advice, a web-based dashboard for health care professionals (HCPs) to monitor patients’ adherence and symptoms, and motivational interviewing (MI) teleconsultations delivered by oncology nurses and pharmacists at baseline and weeks 1, 4, 8, and 12. Primary outcomes were the patients’ acceptance of SAMSON, measured by the Unified Theory of Acceptance and Use of Technology at 12 weeks, and study feasibility, measured by predefined rates of recruitment, randomization, retention, intervention adherence, and outcome assessment completion. Secondary outcomes were comparison of MA and clinical self-assessments through online questionnaires, including adherence, toxicity self-management, anxiety and depression symptoms, and quality of life, measured at baseline and 12 weeks between the 2 arms. Data retrieved from the SAMSON app (Swinburne University of Technology) was analysed for task completion.

**Results:**

A total of 33 patients (79% of those who were approached) consented to participate in the trial. Of those, 31/33 (94%) completed baseline surveys and were randomized to SAMSON (15/31) and control arms (16/31). Of 31 patients, 28 (90%) completed the 12-week surveys (12 SAMSON and 16 control). Overall, patients rated the SAMSON solution as highly acceptable (13/15, 87% app usage; 14/15, 93% MI teleconsultation delivery). They reported that SAMSON was easy to use (10/12, 83%) and helpful in improving their MA (6/12, 50%). All study HCPs reported the SAMSON solution was helpful in supporting patients’ MA. Patients completed an average of 99 tasks over the 12-week study period (71% of scheduled tasks). Most patients (10/12, 83%) completed all 5 scheduled consultations. All study feasibility measures were higher than the predefined upper thresholds, except the rate of patients’ responses to medication reminders.

**Conclusions:**

The results demonstrated that the SAMSON solution is acceptable, usable, and useful for oncology HCPs and patients with cancer. The SAMSON solution is feasible in real-life oncology settings. Our next steps involve refining the SAMSON solution based on participants’ feedback, conducting a large-scale randomized controlled trial to evaluate its clinical and economic effectiveness, and exploring potential commercialization.

**Trial Registration:**

Australian New Zealand Clinical Trials Registry ACTRN12623000472673; https://www.anzctr.org.au/Trial/Registration/TrialReview.aspx?id=385728

**International Registered Report Identifier (IRRID):**

RR2-10.1136/bmjopen-2023-079122

## Introduction

Increasingly, cancer is being treated with self-administered medications [1,2], often involving long and complex treatment regimens [2-4]. A patient’s ability to adhere to medications throughout the treatment period is central to the successful delivery of self-administered anticancer regimens [5,6]. However, medication adherence (MA) in cancer is low [7] and often decreases over time [8]. The MA rate is particularly low in hematological cancers, with a variation between 6% [9] and 53% [10]. Improving MA in patients with cancer is crucial, as evidence shows that nonadherence is associated with low survival rates, disease progression, as well as increased health care use and costs [6,11,12].

Given the importance of MA in cancer, there has been an increase in the number of MA interventions in recent times, especially digital interventions [13,14]. With the boom of technology, for instance, smartphones [15], digital health solutions promise more advantages in terms of improved clinical outcomes, and cost efficiency, and they are increasingly accepted by patients [16]. However, evidence on the quality and effectiveness of available MA interventions in cancer remains sparse [17,18]. Our most recent systematic review showed that MA interventions that have multicomponents that are theory- and evidence-based and rigorously designed and evaluated are more likely to be effective [18].

Based on findings from the literature review and the need to address the medication nonadherence problem in cancer, we developed Safety and Adherence to Medications and Self-care Advice in Oncology (SAMSON), a multicomponent digital solution to improve MA. Individual components of the SAMSON solution were co-designed and rigorously developed based on evidence and largely informed by behavioral science research and design science research methodology. The solution comprises 2 components. The first is a smartphone app (SAMSON app) involving individually tailored phone alerts and real-time advice for side effect self-management [19] and a web-based dashboard where patients can track their adherence performance and health care professionals (HCPs) can manage patients’ profile, view their adherence performance and survey responses, and manage their medication schedules and related side effects. The second is a motivational interviewing training platform (MITP) to train HCPs in motivational interviewing (MI) techniques to support patient adherence and side effect self-management [20]. After being co-designed and developed, the SAMSON app was tested on end users, specifically patients with hematological cancer [19], and the MITP was tested on HCPs [20]. We hypothesized that the combination of these 2 components (the SAMSON solution) would be broadly acceptable to patients, practically feasible in a busy clinical practice, and potentially effective in improving MA for patients with cancer.

## Methods

The CONSORT (Consolidated Standards of Reporting Trials) EHEALTH checklist V 1.6.1 [21] was used to report the present study (Multimedia Appendix 1).

### Study Design

We aimed to test the acceptability, feasibility, and potential effects of the SAMSON solution on 30-50 patients with hematological cancer through a 2-armed, unblinded, 12-week, pragmatic pilot randomized controlled trial (RCT) [22]. After providing written consent (Multimedia Appendix 2) and completing baseline questionnaires (Multimedia Appendix 3), participants were randomized to either the intervention group (SAMSON solution) or the control group (usual care) at a 1:1 ratio. After 12 weeks, they completed end-of-study surveys (Multimedia Appendix 3).

### Randomization

Using a computer-generated randomization chart, a permuted block randomization of size 4 was used to ensure an even balance of patients in each group throughout the study period. The allocation schedule was generated by a statistician who was blinded to the participants to prevent any predictability when randomizing participants to intervention or control [23].

### Patients and Eligibility

Patients were recruited from a metropolitan specialized cancer hospital in Melbourne, Australia, between August 2023 and February 2024. Eligible patients were adults (more than 18 years old), diagnosed with hematological cancer, scheduled to commence oral anticancer medicines (OAMs) or commenced the medication for less than 12 months, willing to have OAMs dispensed at the hospital for the duration of the trial, able to communicate in English, and had access to the internet, a smartphone or computer, and telehealth.

### Recruitment

Patients were identified by study site nurses, pharmacists, or treating consultants, who were informed about the study and eligible criteria. At their scheduled consultation, they were asked if they would like their details passed on for contact by the research team. If the patient agreed, they were referred to the study RC. Then, the RC contacts the patient for screening and a comprehensive informed consent process, either in person or online.

### Intervention

#### SAMSON Solution

Patients allocated to the intervention group received the SAMSON solution in addition to their usual care at Peter MacCallum Cancer Centre (PMCC). They were instructed by the RC on how to install the SAMSON app on their smartphone and received login details with a protected password as well as the SAMSON app user manual. Patients’ personal and clinical information, such as diagnosis and treatment, extracted from the hospital’s electronic medical record system (EMR), was entered in the SAMSON web-based dashboard by the RC and made available in the smartphone app.

Patients were informed that they would receive a teleconsultation (either via phone or telehealth) from a hospital clinical pharmacist in the first 3 days after enrolling in the study, and a maximum of 4 follow-up teleconsultations from a hospital clinical nurse on weeks 1, 4, 8, and 12 of the study. These teleconsultations were on top of the usual care at the hospital. All teleconsultations followed predefined structures and were delivered by hospital clinical nurses and pharmacists who were previously trained in MI using the developed MITP. The initial consultation took 30-60 minutes, aiming to provide education on the OAM(s) that the patient received and the importance of adherence, support the patient in making decisions regarding their medication-taking schedule, and identify and document possible risks and barriers to MA.

Based on the agreed medication-taking schedule, individualized daily medication reminders and weekly side effect surveys were set up in the SAMSON backend platform using the web-based dashboard user interface, so patients can receive them in the installed smartphone app. Medicine information and side effect self-care advice, developed by experienced oncology pharmacists based on available reliable resources and clinician review, and approved by PMCC’s Human Research Ethics Committees (HREC), were populated in the SAMSON backend platform using the web-based dashboard. Patients were asked to respond to daily medication reminders and weekly side effect surveys, as well as review self-care advice in the smartphone app. Data on patients’ adherence and drug toxicity collected through the SAMSON solution were stored centrally on a secured server, then aggregated, analyzed, and uploaded onto the web-based dashboard so that study nurses and pharmacists could monitor patients’ adherence and symptoms. These data were also used by HCPs to tailor their teleconsultations with patients. Patients used the SAMSON app throughout the 12-week period of the study.

The follow-up structured teleconsultations (15-30 minutes in length) aimed to check the patient’s understanding of diagnosis, symptoms, self-care strategy, and medications; further explore the patient’s facilitators and barriers to MA; motivate the patient’s adherence, strengthen their medication self-management skills, and change patient’s nonadherence behavior by using MI skills. The quantity and length of these consultations were tailored to the individual patient’s need and adherence status.

Intervention nurses and pharmacists had more than 5 years of clinical experience in providing oncology care and successfully completed MI training via the MITP. They were also equipped with instruction manuals on how to conduct teleconsultation and use the SAMSON web-based dashboard. Brief notes were produced and recorded in hospital electronic medical records, as well as sent to the patient at the end of the consultation session.

#### Usual Care

Patients who were allocated to the control group received usual care. The usual care at the hospital consisted of a clinician consultation, an initial in-person pharmacist consultation (often 5-10 minutes in length), and a phone call follow-up from a clinical nurse within 1-2 weeks after commencing medication.

### Measures

Demographic was completed at baseline (t_0_). Patients’ personal and clinical information was collected from the hospital’s electronic medical record system. The study’s primary outcomes included the patients’ acceptance of SAMSON, measured by the Unified Theory of Acceptance and Use of Technology (UTAUT) [24,25] at 12 weeks (t_1_), and study feasibility, measured by predefined rates of recruitment, randomization, retention, intervention adherence, and outcome assessment completion [26,27]. Secondary outcomes included MA, self-reported adherence, toxicity self-management, anxiety, depression and symptoms, and quality of life. Outcomes were assessed at baseline (t_0_) and at the end of week 12 (t_1_), except MA measured in week 16. All survey data collection (Multimedia Appendix 3) was done through (Research Electronic Data Capture) REDCap (Vanderbilt University) [28].

### Primary Outcome Measures

#### Acceptability

The UTAUT questionnaire was adapted to assess determinants of HCPs’ and patients’ acceptance and use of the SAMSON solution, including 5 dimensions: performance expectancy, effort expectancy, social influence, facilitating conditions, and behavioral intention [25]. Patients were asked to rate their satisfaction with the SAMSON solution on a 5-point Likert scale. Participants were also invited to provide free-text feedback and suggestions on the solution.

#### Feasibility

A traffic light approach [29] (Table S1 in Multimedia Appendix 4) was used to determine the feasibility success at 3 levels: (1) feasible (above the upper threshold), (2) infeasible (below the lower threshold), and (3) protocol revision (between the 2 thresholds). The thresholds were defined in consultation with the Steering Committee and based on the literature of similar studies. Informal discussions with study personnel were attempted to obtain feedback on the feasibility.

### Secondary Outcome Measures

MA was measured by medication refill adherence (MRA) [30] collected from pharmacy dispensing data. MRA was defined as a percentage calculated from the total days’ supply divided by the number of days of study participation and multiplied by 100. In this study, the patient was considered as optimal adherence if their MRA was≥90%.

Self-reported adherence (Adherence Starts with Knowledge 12 [ASK-12]) was measured [31]; this measure included 12 items in 3 subscales: adherence behavior, health beliefs, and inconvenience or forgetfulness.

Toxicity self-management was measured with the Patient Activation Measure-Short Form [32,33], a 13-item self-report measure assessing the patient’s knowledge, skills, and confidence in the self-management of their disease and related symptoms.

Anxiety, depression, and symptoms were measured by the Patient-Reported Outcomes Measurement Information System (PROMIS) [34] to assess depression, anxiety, pain interference, fatigue, sleep disturbance, and physical function.

Quality of Life was measured by Functional Assessment of Cancer Therapy-General (FACT-G) [35], which is a 27-item self-report scale measuring the quality of life of patients currently undergoing cancer treatment.

### Analysis

Descriptive statistics were used to summarize participant characteristics across study arms, and differences in baseline attributes were assessed using *t* tests or chi-square tests as appropriate.

SAMSON acceptability was analyzed thematically. The UTAUT aims to examine individual quality dimensions, which means it is a suite of scales rather than one quality measure; therefore, adding up the overall scale of the questionnaire is not suitable. Results of UTAUT surveys were summarized for each of the 5 dimensions with the percentage of patients endorsing Likert scale ratings of 3 (disagree, neither agree nor disagree, and agree). Free text answers to UTAUT questionnaires were narratively summarized to gain further insight into acceptability.

Feasibility was determined based on the traffic light approach. Recruitment feasibility was assessed by the number of patients recruited (consented) divided by the number of patients who were approached to join the study. Randomization feasibility was assessed by the number of patients who were randomized divided by the number of patients who consented. Retention in both arms of the study was assessed by the number of patients who remained at the end of the study divided by the total number of patients who consented to join the study. We also tracked intervention adherence, for example, the percentage of patients who completed tasks on the SAMSON smartphone app and received MI teleconsultations. Compliance data for survey completion was calculated as the percentage of patients who completed surveys at t_0_ and t_1_*t_1_* out of the total patients in the study at these time points.

Secondary outcomes were analyzed using linear regression. The dependent variable was the outcome at follow-up and the independent variables were arm assignment and the outcome at baseline. Standard checks for normality and homoscedasticity of residuals were conducted for each outcome. All analyses were conducted using StataNow 18 (StataCorp) [36].

### Ethical Considerations

A steering committee—which included chief investigators; experts in the fields of digital health, information technology, nursing, pharmacy, psychology, and oncology; and a patients’ representative—was formed to provide support for the study. This study was approved by the HRECs of PMCC (number HREC/95332/PMCC) and Swinburne University of Technology (number 20237273-15836) (Multimedia Appendices 5 and 6). Patients reviewed study details and indicated their consent using e-consent forms. Patients were encouraged to contact the study team if they had any questions or concerns. Patients’ personal and health information in the SAMSON app was encrypted in transit and stored in a secure server at Swinburne. Study team access to patient’s data on the SAMSON web-based dashboard was password protected and limited to the research coordinator (RC) and 4 study nurses and pharmacists. All data analyses were conducted on deidentified data. Patients received a US $31 gift voucher if they completed all surveys in the study.

## Results

### Patient Characteristics

**Figure 1:**
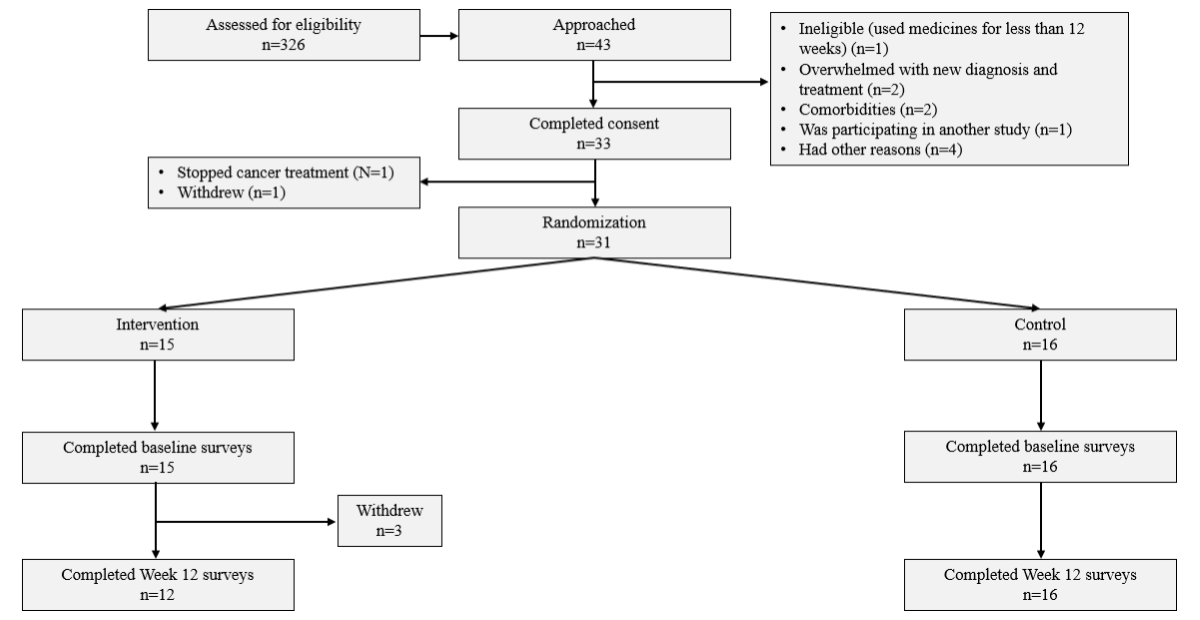
CONSORT (Consolidated Standards of Reporting Trials) diagram of Safety and Adherence to Medications and Self-care Advice in Oncology (SAMSON) pilot randomized controlled trial.

Among 42 patients who were approached, 33/42 (79%) consented to participate, and 31/33 (94%) completed baseline (t_0_) surveys. Of those who completed baseline surveys, 3/31 patients (all from the intervention arm) withdrew from the study (10 %) due to the burden of the disease. All the remaining 28 patients (100%) completed week-12 surveys (12 intervention and 16 control) (Figure 1).

Demographics of randomized patients are provided in Table S2 in Multimedia Appendix 7. There were no significant differences in participants’ demographics between the 2 arms.

### Acceptability Results (Primary Outcome 1)

#### Overview

Out of 15 intervention arm patients, 13/15 (87%) installed the SAMSON app on their phone for use (2 patients withdrew). All 15 patients in the intervention arm received the initial pharmacy teleconsultation. Most patients (14/15, 93%) received at least 1 nurse teleconsultation, and 10 of them (10/15, 67%) completed all 4 scheduled nurse teleconsultations.

Data retrieved from the SAMSON app showed moderate engagement among participants. Patients completed an average of 99 app tasks (including responses to daily medication reminders and weekly side effect surveys) over the 12-week study period, which accounted for 99/140, 71% of scheduled tasks. A total of 36 severe episodes of symptoms were reported.

**Figure 2:**
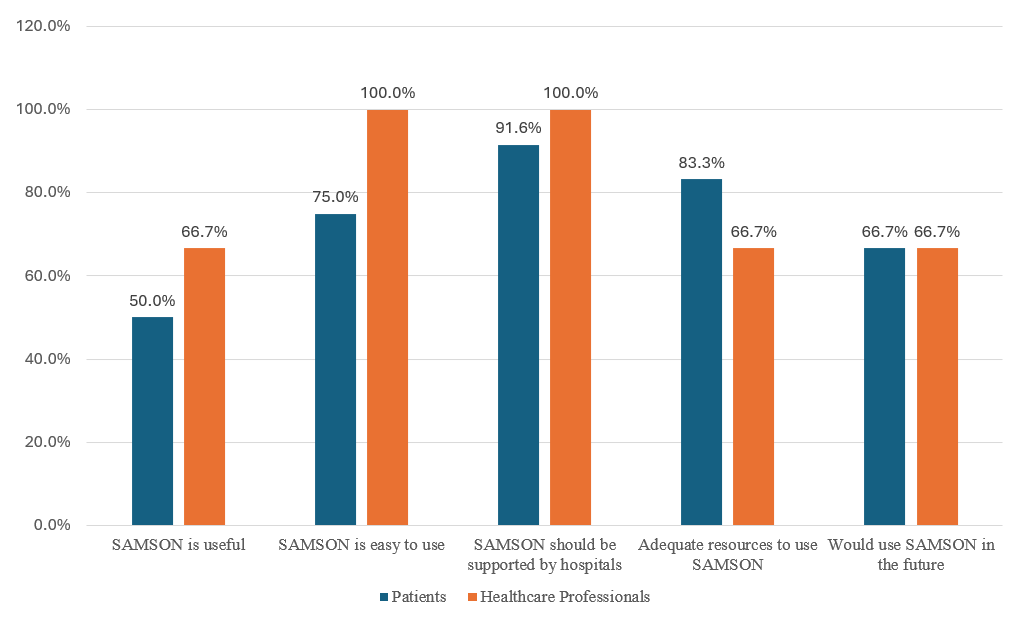
Patients’ and health care professionals’ opinions about Safety and Adherence to Medications and Self-care Advice in Oncology (SAMSON) solution (n=12 and n=3, respectively).

Out of 15 patients, 12/15 (80%) and 3 study HCPs (100%) completed the UTAUT surveys. A summary of patients’ and HCPs’ responses to the UTAUT questionnaire is presented below, with full details in Multimedia Appendices 8 and 9. Figure 2 presents patients’ and HCPs’ key opinions about the SAMSON solution.

#### Performance Expectancy

Half of the patients (6/12) in the intervention arm reported that their MA could improve with the help of the SAMSON solution. Patients used different MA-supporting mechanisms provided by SAMSON solution, including prompting reminders (7/12, 58%), disease and treatment education (10/12, 83%), improving confidence in treatment (6/12, 50%), and improving side effect self-management skills (9/12, 75%). Despite this, 3/12 patients (25%) found the SAMSON app component was not that helpful. Of those, 2 were using another commercial MA app with greater functionality. Some patients (4/12, 33%) reported functional issues of the app occurring in a short period of time (1-3 days), for example, medication reminders were not delivered or responses to reminders were not saved properly.

Of the 3 HCPs participating in the trial, 2 found the SAMSON solution useful in their job. All HCPs agreed that SAMSON could enable their 2-way communication with patients which would increase their ability in supporting patient treatment adherence. However, 1 HCP suggested that the SAMSON dashboard’s visual design could be improved to be more appealing. HCPs’ comments on the time required for MI teleconsultations were controversial: some suggested that the scheduled time was good, while it was reported as quite long for another, which might be a constraint in clinical practice.

#### Effort Expectancy

The SAMSON app was found to be easy to use by most patients (10/12, 83%). Its presentation was clear, and the content was easy to understand (11/12, 92%). Most participants could easily navigate the app (10/12, 83%), yet 1 (1/12, 8%) struggled in responding to surveys in the app. Some issues with the app’s functionality were reported, for example, the medication reminders’ time was not automatically updated after the daylight saving time changed, and the app was frozen sometimes, which affected patients’ responses to reminders.

All HCPs reported that SAMSON was quite easy to use. However, some issues with the SAMSON web-based dashboard were reported, for example, it did not allow more than 1 HCP to monitor the patient, so all study HCPs had to share 1 account. Two (2/3, 67%), HCPs found implementing MI consultations quite challenging in practice due to time constraints.

#### Social Influence

Out of 12 patients, 5 (41.6%) thought that their family and friends would support their use of SAMSON, while most of them (10/12, 83%) thought that other patients with cancer would find SAMSON valuable. Almost all patients (11/12, 92%) desired SAMSON to be available for use in cancer hospitals.

All HCPs commented that their colleagues would find the SAMSON valuable and desire to receive the hospital’s support in implementing SAMSON in daily practice.

#### Facilitating Conditions

Most participants were confident that they had adequate knowledge and resources (10/12, 83%) to use SAMSON and could access support when needed (9/12, 75%). Nevertheless, 2/12 patients (17%) reported their difficulties when dealing with some technical issues when using the app.

Despite all HCPs reporting that they had adequate knowledge to apply MI techniques in teleconsultations, 1/3 (33%) wished to have more training resources on the SAMSON app.

#### Behavioral Intention

Most patients (9/12, 75%) felt confident using the SAMSON app. They would want to continue using SAMSON after the study finishes (8/12, 67%) or recommend it to their peers (7/12, 58%). The 3 patients (25%) who were using another commercial MA app with more functionalities would prefer to receive MI teleconsultations alone. Cost was reported as an important factor influencing the intention to use SAMSON by the majority of patients (8/12, 67%).

Regarding the open-ended questions in the UTAUT survey on experience and perception of the SAMSON solution, patients valued the SAMSON smartphone app, because it was “easy to use and prompted reminders” (P31), “placed all medication resources in one place” (P16), “informative and helpful” (P25), and provided “help quickly if [the patient has] any queries” (P30). The “side-effects” tab within the app, providing self-care advice, was reported to make the patient “feel more secure in managing medication regimens” (P2), which could result in high adherence—as one patient commented, “I didn’t miss a dose” (P7). The number of teleconsultations, duration, and quality were reported by most patients as good or “perfect” (P3) and “at the right length of time and allowed me [the patient] to ask all questions I need while allowing the nurse to gather all information” (P2); however, 1 mentioned it was “too long” (P31). Patients provided some helpful suggestions to improve the convenience of using the SAMSON smartphone app, including fixing glitches, combining all medications scheduled at the same time in 1 reminder (rather than separate reminders), having more options for responding to reminders, and more attractive presentation.

Over 60% of study HCPs felt confident in delivering SAMSON to patients in the trial and would like to continue using it in the future. All of them would introduce the solution to their peers to use.

### Feasibility Results (Primary Outcome 2)

The study recruitment rate was 33/42 (79%). Most participants went through the information and consent process via phone, except 4 were recruited on-site. The randomization rate was 31 of 33 (94%). The retention rate was 28 of 33 (85%). The RC conducted phone calls or in-person check-ups at least 2 times during the 12-week study. All recruitment, randomization, and retention rates were higher than the upper threshold.

A total of 13 intervention arm patients (13/15, 87%) used the SAMSON app. The proportions of responses to medication reminders and side effect surveys compared to the scheduled tasks during the 12-week study period were 1061 of 1541 (68.9%) and 128 of 141 (90.8%), respectively. The unmet threshold of responses to medication reminders could be due to the app’s functional issues as reported earlier. In total, 65 MI teleconsultations were delivered from August 2023 to June 2024, of which 35 of 65 (54%) were conducted on time as scheduled, 26 (40%) were later than scheduled, and 4 (6%) were missed. The average length of initial pharmacy consultations was 40 minutes, while nurse follow-up consultations were 40 minutes on average. Reasons for delayed and missed consultation sessions were mostly on the patient side, including not showing up at the appointment (25/30, 83%), not responding to HCP phone calls (4/30, 13%), or international travel (1/30, 3%). Only 2 sessions (7%) were delayed, because the HCP could not match their work schedule. Of the 12 intervention arm patients who were retained until the end of the study, 10 of 12 (83%) received all 5 scheduled teleconsultations, 1 (8%) received 4 teleconsultations and 1 (8%) received 2 teleconsultations.

Baseline surveys were completed by 31/33 (94%) patients. All who stayed until the end of the study (28/28, 100%) completed week-12 surveys. Participants completed all surveys online without any need for support. An alert email was sent by RC to all participants a few days before the survey due date. However, over one-third of participants (10/28, 36%) only completed surveys after being reminded. Details of feasibility results are presented in Table S3 in Multimedia Appendix 10.

### Preliminary Efficacy Results (Secondary Outcomes)

#### Medication Refill Adherence

Of 28 patients who completed the study, 25/28 (89%) had a 100% adherence rate and 3/28 (11%) had over 95% adherence rate. There is no difference in the proportion of patients who had optimal adherence (MRA ≥90%) between the intervention and control groups (100% for both groups).

The mean and 95% CI of ASK-12, Patient Activation Measure-Short Form, PROMIS, and Functional Assessment of Cancer Therapy-General at baseline and week 12 are shown in Table S4 in Multimedia Appendix 11.

## Discussion

### Principal Findings

In this study, we aimed to test the acceptability, feasibility, and preliminary efficacy of a multicomponent MA solution, SAMSON, to help patients with cancer improve their adherence to OAMs and self-manage their physical and emotional symptoms. Overall, patients and oncology HCPs rated SAMSON as highly acceptable, usable, and useful. These high levels of user satisfaction evidence that the solution meets the various needs of support among patients with cancer to medically adhere to and manage side effects at home [37], as well as the needs of oncology HCPs for a practical and tailored MI training, and a means to regularly monitor and provide ongoing support to patients’ MA [20]. Moreover, qualitative findings suggest that SAMSON has the potential to help patients in MA and symptom self-management, as well as to assist HCPs in monitoring and supporting patients’ adherence. The results of the study help to address the gap of knowledge and the need in oncology practice [17,18] by providing evidence of the high quality and potential effect of a digital multicomponent MA solution.

Regarding SAMSON’s feasibility, we noted a high level of engagement with the SAMSON solution in terms of the overall tasks completed by patients on the SAMSON smartphone app and the MI teleconsultations completed by both HCPs and patients. The high acceptability and feasibility of SAMSON is a result of several factors. First, co-design and rigorous design framework were applied to develop SAMSON [22]. By involving end users and stakeholders throughout all design stages of the intervention, co-designing helped to improve the solution’s acceptability, desirability, and usability [38,39]. The use of design frameworks, for example, design science research methodology in this study, could enhance the artifact’s design, which is crucial in digital intervention development, and improve its acceptance, usage, and efficacy [40]. Furthermore, the design framework usage can improve the rigor and translatability of research, which is currently limited or poorly reported in the development of available MA interventions in cancer [18]. Second, after development, individual components of SAMSON were successfully tested by end users on their acceptability, usability, and usefulness [19,20]. To the best of our knowledge, SAMSON is the first comprehensive digital MA solution in cancer that is co-designed, theory-based, evidence-based, and rigorously developed and tested.

Although overall acceptance and feasibility were relatively high, some HCPs and patients in the trial reported several technical issues with the SAMSON app and desired better visualization, more functionality, and further instructions and training. This feedback will be used to further improve the solution in the future. A couple of study HCPs were concerned about the time required for MI consultations. Time constraints have been reported as one of the barriers to MI implementation in clinical practice [41]. It is noted that in this study, SAMSON was delivered as an add-on service on top of the hospital’s usual care, which required extra time and effort from busy HCPs. In addition, delivering MI consultations to promote adherence was a newly acquired skill by the study HCPs, thus, they required more time to master this approach. A holistic approach, including the hospital’s mechanisms to provide continuous MI monitoring and training, as well as support (both in terms of skills and resources) to facilitate and maintain HCPs’ confidence and motivation in using this skill set can be a solution to tackle MI implementation barriers [41,42].

The overall recruitment, randomization, retention, and data collection compliance rates were higher than the preset upper thresholds. A high attrition rate is generally one of the major concerns in digital health RCTs compared to RCTs testing more “traditional” interventions [43]. However, the high recruitment rate and the low rate of loss to follow-up in this trial indicate that the SAMSON solution and online pragmatic RCT design are feasible in busy oncology clinical settings, as long as appropriate methodological strategies are applied. Specifically, in this study, clinical nurses and pharmacists from the Hematology Department were recruited and funded to support study recruitment and deliver teleconsultations. The RC dedicated additional time to building rapport with participants and consistently assisted them through all processes of the trial (consenting, baseline assessment, randomization, app installation, technical training and support, and outcome assessments). In addition, different channels of communication, for example, emails, phone calls, and SMSs were used to follow-up and motivate participants throughout the study. The importance of check-ups and follow-ups postrandomization in trials of digital interventions, where participants are required to have certain levels of digital literacy to perform tasks of the intervention [44], has been emphasized in several studies [45]. Future digital trials may benefit from these recruitment and retention strategies.

In this trial, a combination of techniques was used to measure MA, including self-report measures and prescription refill reports. Although triangulation of measurements could improve the accuracy of adherence [46], its interpretation needs to be cautious. The MRA using pharmacy computer records, which is objective [47], does not guarantee that all dispensed medications were consumed by patients. The self-reported adherence survey (ASK-12) is simple, but often subjective and more about barriers to adherence than actual adherence status [31]. Future studies should consider using high-accuracy methods, such as the Medication Event Monitoring System [5], to measure MA.

This study has some limitations. Like many digital health studies, our sample was predominantly Caucasian and highly educated [48,49]. Consequently, the findings may not be representative of patients who are non-Caucasian or have lower socioeconomic status, who might face higher barriers to adherence and could potentially benefit more from the SAMSON solution than their Caucasian or higher socioeconomic status peers [50,51]. Participants were recruited from the Hematology department at PMCC, one of Australia’s leading oncology hospitals, and were prescribed only 1 oral anticancer regimen. Therefore, the results may not be generalizable to those who use multiple anticancer medications or receive care in low-resource oncology settings. A more targeted recruitment strategy focusing on underserved cancer patient populations with other types of cancer in various levels of oncology care institutions is warranted.

### Conclusion

Before undertaking this pilot trial, both components of the SAMSON solution were co-designed and developed based on evidence and theory, and then individually tested on target users. The results of this study confirmed that SAMSON is acceptable, usable, and useful for both HCPs and patients with cancer. Both the SAMSON solution and the pragmatic RCT design are feasible in real-life oncology settings. These findings are very encouraging, given the numerous challenges in applying RCT as an evaluation design for digital health interventions [52]. Our next steps will involve refining the SAMSON solution based on participants’ feedback from this study and conducting a full RCT to evaluate its clinical and economic effectiveness.

## Appendix

Multimedia Appendix 1 CONSORT-eHEALTH checklist (V 1.6.1).

Multimedia Appendix 2 Participant information statement and consent form.

Multimedia Appendix 3 Survey booklet.

Multimedia Appendix 4 Table S1. Thresholds for traffic light approach to feasibility.

Multimedia Appendix 5 Ethics approvals.

Multimedia Appendix 6 Ethics approvals.

Multimedia Appendix 7 Table S2. Participant demographics by arm.

Multimedia Appendix 8 Unified Theory of Acceptance and Use of Technology results of patient participants.

Multimedia Appendix 9 Unified Theory of Acceptance and Use of Technology results of health care professional participants.

Multimedia Appendix 10 Table S3. Study feasibility results.

Multimedia Appendix 11 Table S4. Differences and change from baseline to week 12 between arms, analysis of covariance test.

## Abbreviations

ASK-12: Adherence Starts with Knowledge 12
HCP: health care professional
HREC: Human Research Ethics Committee
MA: medication adherence
MI: motivational interviewing
MITP: motivational interviewing training platform
MRA: medication refill adherence
OAM: oral anticancer medicine
PMCC: Peter MacCallum Cancer Centre
PROMIS: Patient-Reported Outcomes Measurement Information System
RC: research coordinator
RCT: randomized controlled trial
SAMSON: Safety and Adherence to Medications and Self-care Advice in Oncology
UTAUT: Unified Theory of Acceptance and Use of Technology

## Data Availability

The datasets generated and analyzed during this study are not publicly available but are available from the corresponding author on reasonable request.
