# Supplementary material for: Digital Solution to Support Medication Adherence and Self-Management in Patients with Cancer (SAMSON): Pilot Randomized Controlled Trial
Source: JMIR Form Res. 2025 Feb 19;9:e65302. doi: 10.2196/65302 (PMC11888109; doi:10.2196/65302)
Supplement: Multimedia Appendix 2 [file formative_v9i1e65302_app2.docx]

**Participant Information Sheet/Consent Form**

| **Title** | Pilot randomized controlled trial (RCT) to test the acceptability, feasibility, and potential efficacy of the SAMSON (Safety and Adherence to Medications and Self-care Advice in Oncology) intervention solution  (Short title: SAMSON pilot RCT). |
| --- | --- |
| **Project Sponsor** | Peter MacCallum Cancer Centre,  Digital Health Cooperative Research Centre,  and Swinburne University of Technology. |
| **Principal Investigator** | Professor Penelope Schofield |

1. **Introduction**

You are being invited to take part in a research project that tests the SAMSON (Safety and Adherence to Medications and Self-care Advice in Oncology) intervention solution because you have been prescribed oral (taken by mouth) medicines for your cancer treatment.

This Participant Information Sheet/Consent Form tells you about the research project. Please read this information carefully. Ask questions about anything that you don’t understand or want to know more about.

Participation in this research is voluntary. If you don’t wish to take part, you don’t have to. If you decide to take part and later change your mind, you are free to withdraw from the project at any stage. Your decision whether to take part or to not take part or to take part and then withdraw, will not affect your relationship with Peter MacCallum Cancer Centre (Peter Mac) or Swinburne University of Technology (SUT).

If you decide you want to take part in the research project, you will be asked to sign the consent section and you will be given a copy of this Participant Information Sheet/Consent Form to keep.

**2. What is the purpose of this research?**

The SAMSON is a digital platform to support patients who are taking oral cancer medications by improving their medication adherence to the treatment and reducing any medication errors. Medication adherence means taking your medication as prescribed by your doctor. Medication errors are problems that can occur when taking medication (for example, your medication interacts with other medications you already took so that it doesn’t work as it should).

The SAMSON trial will assess the acceptability, feasibility, and potential efficacy of the SAMSON solution. This research project has been initiated by Professor Penelope Schofield and it is supported by research funding from PeterMac, Digital Health Cooperative Research Centre, and SUT. The Research Coordinator, a PhD student, Thu Ha Dang, will implement this project with support from the research team. The results of this research will be used by Thu Ha Dang to obtain her Doctor of Philosophy degree.

**3.** **What does participation in this research involve?**

***3.1. What are the requirements to participate in this research project?***

To be able to participate in this research project, you will need to meet all of the following requirements:

1. A confirmed diagnosis of any-stage haematological, lung, or melanoma cancer;
2. Scheduled to commence oral anti-cancer medicine(s) as part of routine care or commencing treatment/currently treated with the medication for less than 12 months;
3. Willing to have oral anti-cancer medicine(s) dispensed at Peter Mac;
4. Over 18 years old;
5. Proficient in English; and
6. Accessibility to the internet, a smartphone (and computer), and/or telehealth.

If you decide to participate, the research coordinator will inform your medical team of your decision to take part in this study.

***3.2. Which group will I be allocated to?***

You will be participating in a Randomised Controlled Trial (RCT). This means we will compare a group of people given the SAMSON intervention (the intervention group), with a group of people who will receive the standard level of care at Peter Mac (the non-intervention group). To try to make sure the groups are the same, each participant will be assigned to either the intervention or non-intervention group by chance (at random). This means that you will have an equal chance of being assigned to either group. If you are assigned to the intervention group, you will receive the SAMSON intervention. If you are assigned to the non-intervention group, you will receive the standard level of care from your medical team.

***3.3. What are the intervention and non-intervention groups?***

People in both groups will be asked to complete some online questionnaires at the start, during, and at the end of the 16-week study period. You will be emailed an online link to access these questionnaires on your computer. In total, these questionnaires will take roughly 30-45 minutes to complete, and will look at how often you take your medication, any symptoms you might experience, and your mood and quality of life. Basic demographic and clinical questions will be also asked (for example, your sex, age, occupation, and the time since your diagnosis). Questionnaires in hard copy will also be available should the need arises.

All of the information we collect about you will be de-identified once it has been collected. This means that your name will be replaced by a unique participant code so that your personal identity is not connected with your data. All of your information that has been collected using a unique participant code (paper-based, recorded, and online) will be stored in a secure location to maintain your confidentiality. Identifying information, such as your name and contact details will be stored in a separate password-protected file and only accessed by the principal investigator or research coordinator.

There are no additional costs associated with participating in this research project. You will receive a gift voucher of A$50 for your time spent completing all surveys as requested in sections 3.4 and 3.5.

If you wish to be contacted for future research projects on medication adherence, please indicate this in the consent form and we will contact you when they are available.

***3.4. The intervention group***

If you are allocated to the intervention group, then you will receive:

- An initial face-to-face or online consultation (30-60 minutes) with a pharmacist to talk about your medication (right after you are enrolled in the study),
- Four teleconsultations with an intervention nurse or pharmacist to support you in taking your medication (in weeks 1, 4, 8, and 12). Each of these appointments will take between 15-30 minutes and will be arranged at a time that suits you. A written summary of each teleconsultation with the nurse/pharmacist will be sent to your email address.
- SAMSON mobile application (app) that will:
- Send you daily medication reminder notifications,
- Send you a weekly survey to monitor any side effects that you may be experiencing in relation to your oral medication.

Your treatment team may receive a correspondence informing them of your progress while you are in the study, especially if you report any serious side effects.

When you start on the SAMSON research project, you will be provided with the SAMSON mobile app user manual to install the SAMSON mobile app onto your smartphone. You will be given a password and username to access the SAMSON system. A member of the research team will assist you to download the SAMSON app and brief you on how to use the SAMSON app and web page. You will have your medication reminders programmed into the app for a time that suits you. These times can be changed if needed. You will also be shown how to access your information on how regularly you take your medication, as well as any side effects that you have reported, on the SAMSON website. This training and information discussion will take place either in person or online, and will likely take around 10-15 minutes to complete.

You will be invited to complete the following questionnaires. The daily responses to medication reminders and weekly side effects surveys will be provided on the SAMSON app. All other surveys can be completed in the link that you will receive via email:

- At the start of the study (after providing consent to participate in the project): a demographic survey
- Week 1 to week 12: responses to daily medication reminders and weekly side effects survey (provided on the SAMSON app)
- Week 1:
- Self-report adherence (ASK-12)
- Toxicity self-management (PAM-SF)
- Anxiety, depression, and symptoms (PROMIS)
- Quality of life (FACT-G)
- Week 12:
- Self-report adherence (ASK-12)
- Toxicity self-management (PAM-SF)
- Anxiety, depression and symptoms (PROMIS)
- Quality of life (FACT-G)
- A survey (UTAUT) for your feedback on how useful and appropriate the SAMSON solution was in helping you to take your medication as advised, and in helping you to self-manage any side effects that you may have experienced. You will also be asked whether you have any suggestions about ways to improve the SAMSON.

***3.5. The control group***

If you are allocated to the intervention group, then you will receive usual care at Peter Mac and be invited to complete the following questionnaires:

- At the start of the study (after providing consent to participate in the project): a demographic survey
- Week 1 and 12:
- Self-report adherence (ASK-12)
- Toxicity self-management (PAM-SF)
- Anxiety, depression, and symptoms (PROMIS)
- Quality of life (FACT-G)

**4.** **What are the possible benefits of taking part?**

By participating in this study, you are helping us to test the SAMSON digital solution. You may find that the SAMSON app and additional healthcare professional consultations are helpful for you in managing your medication treatment.

**5. What are the possible risks and disadvantages of taking part?**

There may have some disadvantages to partaking in this research project, e.g. additional time for study follow-up consultations and time spent (about 30-45 minutes in total) to complete the surveys. Your responses to these surveys will not be monitored by your care team at Peter Mac. If you become upset or distressed as a result of your participation in this study, please raise them to the researcher. The researcher will be able to arrange for counseling or other appropriate support. The researcher will also report this to your treating team at Peter Mac. Any counseling or support will be provided by staff who are not members of the research team.

**6. What if I withdraw from this research project?**

If you do consent to participate, you may withdraw at any time. If you decide to withdraw from the project, please notify a member of the research team and complete the Withdrawal of Consent form. The research coordinator can be contacted via email address: Ha.Dang@petermac.org.

You should be aware that data collected up to the time you withdraw will form part of the research project results. If you do not want your data to be included, you must tell the researchers this when you withdraw from the research project. Please also note that your data will only be able to be withdrawn prior to data analysis. More information about what happens with your data can be found in section 7.

**7. What will happen to information about me?**

By signing the consent form, you are consenting for the research team to collect and use personal information about you for the purposes of this research project. If you are allocated to the intervention group, your study data collected via the SAMSON app will be identifiable to the intervention nurses/pharmacists at Peter Mac. This is so that they can conduct teleconsultations with you, throughout the study. However, all other information that you provided will be coded and remain confidential, and only the research team will have access to the list that can link your name to your individual data. Any information obtained in connection with the SAMSON research project that can identify you will remain confidential and will only be used for the purposes of this research project. All information will be stored in a locked filing cabinet in the office of the research staff. Electronic information, including information collected via the SAMSON app, will be stored in secure files/ folders/ servers with protected passwords that are only known by the research team. All information will be disposed of as confidential waste after seven years from the date of publication results. Your study data will only be disclosed with your permission, except as required by law.

It is anticipated that the results of this research project will be published and/or presented in a variety of forums. In any publication and/or presentation, information will be provided in such a way that you cannot be identified, except with your express permission.

In accordance with relevant Australian and/or Victorian Privacy and other relevant laws, you have the right to request access to the information about you that is collected and stored by the research team. You also have the right to request that any information with which you disagree be corrected. Please inform either the research coordinator or principal investigator named in section 8 of this document if you would like to access your information.

**8. What happens when this research project ends?**

When the project ends, the SAMSON intervention will be evaluated for further research. This means that the SAMSON intervention will not be available for patient use once the project finishes. You will continue to receive treatment and care from your medical team as usual.

If you wish to obtain a final copy of the research report describing the results of this study, please indicate this in the consent form and we will arrange for one to be sent to you.

**9. Who has reviewed the research project?**

All research in Australia involving humans is reviewed by an independent group of people called the Human Research Ethics Committee (HREC). The ethical aspects of this research project have been approved by the HREC of the Peter MacCallum Cancer Centre (HREC Reference No: HREC/…../PMCC).

This project will be carried out according to the National Statement on Ethical Conduct in Human Research (2007). This statement has been developed to protect the interests of people who agree to participate in human research studies.

**10. Further information and whom to contact**

The person you may need to contact will depend on the nature of your query. If you want any further information concerning this project or if you have any problems which may be related to your involvement in the project, you can contact the research coordinator (Thu Ha Dang) via email address: ha.dang@petermac.org; or the principal investigator (Professor Penelope Schofield) at on (03)9214 4886, email: penelope.schofield@petermac.org.

The details of the Peter MacCallum Cancer Centre complaints person are:

**Complaints contact person**

| Position | Consumer Liaison |
| --- | --- |
| Telephone | (03) 8559 7517 |

If you have any complaints about any aspect of the project, the way it is being conducted or any questions about being a research participant in general, then you may contact:

| Reviewing HREC name | Peter MacCallum Cancer Centre Ethics Committee |
| --- | --- |
| HREC Executive Officer | Ethics Coordinator |
| Telephone | (03) 8559 7540 |
| Email | ethics@petermac.org |

**Reviewing HREC approving this research** **and HREC Executive Officer details**

**Consent Form**

| **Title** | Pilot randomized controlled trial (RCT) to test the acceptability, feasibility, and potential effect of the SAMSON (Safety and Adherence to Medications and Self-care Advice in Oncology) intervention solution; and identify the ‘optimal configuration’ of the intervention  (Short title: SAMSON pilot RCT). |
| --- | --- |
| **Project Sponsor** | Peter MacCallum Cancer Centre,  Digital Health Cooperative Research Centre, and Swinburne University of Technology. |
| **Principal Investigator** | Professor Penelope Schofield |

**Declaration by Participant**

- I have read the Participant Information Sheet, or someone has read it to me in a language that I understand.
- I understand the purposes, procedures, and risks of the research described in the project.
- I have had an opportunity to ask questions and I am satisfied with the answers I have received.
- I freely agree to participate in this research project as described and understand that I am free to withdraw at any time during the project without affecting my future care.
- I understand that I will be given a signed copy of this document to keep.

| Name of Participant (please print) | ____________________________________________ |
| --- | --- |
| Signature  _______________________ | Date  ________________________________________ |

| I would like a copy of the overall study results to be emailed to me once the study is completed. Please note that these results are for the complete study, they are not your individual results. | Yes  No |
| --- | --- |
| I would like to be contacted for future research projects on medication adherence. | Yes  No |

**Declaration by Researcher^†^**

I have given a verbal explanation of the research project, its procedures, and risks, and I believe that the participant has understood that explanation.

| Name of Researcher (please print) | ___________________________________________ |
| --- | --- |
| Signature _______________________ | Date  ________________________________________ |

^†^ An appropriately qualified member of the research team must provide an explanation of, and information concerning the research project.

Note: All parties signing the consent section must date their own signature.

**Withdrawal of Consent**

| **Title** | Pilot randomized controlled trial (RCT) to test the acceptability, feasibility and potential efficacy of the SAMSON (Safety and Adherence to Medications and Self-care Advice in Oncology) intervention solution |
| --- | --- |
| **Short Title** | SAMSON pilot RCT |
| **Protocol Number** | Protocol Version: 1.0 |
| **Project Sponsor** | Peter MacCallum Cancer Centre (Peter Mac),  Digital Health Cooperative Research Centre (DHCRC), and  Swinburne University of Technology (SUT) |
| **Principal Investigator** | Professor Penelope Schofield |
| **Location** | Peter Mac Parkville |

**Declaration by Participant**

I wish to withdraw from participation in the above research project and understand that such withdrawal will not affect my relationships with the researchers, Peter Mac, DHCRC, or SUT. I understand that the research team and relevant study staff will not collect any more information from me, but only collected data that has not been analysed may be withdrawn.

**Name of Participant (printed)**________________________________________________

**Signature**__________________________________ **Date:** _____ / _____ / _____
