# Supplementary material for: Digital Solution to Support Medication Adherence and Self-Management in Patients with Cancer (SAMSON): Pilot Randomized Controlled Trial
Source: JMIR Form Res. 2025 Feb 19;9:e65302. doi: 10.2196/65302 (PMC11888109; doi:10.2196/65302)
Supplement: Multimedia Appendix 3 [file formative_v9i1e65302_app3.docx]

**SAMSON Pilot Randomized Controlled Trial (RCT)**

**SURVEY BOOKLET**

Contents

[**SECTION I. DEMOGRAPHIC SURVEY** 2](#_Toc129190559)

[**SECTION II. POST-INTERVENTION SURVEY FOR HEALTHCARE PROFESSIONALS** 5](#_Toc129190560)

[**SECTION III. POST-INTERVENTION SURVEY FOR PATIENTS** 8](#_Toc129190561)

[**SECTION IV. PATIENT ACTIVATION MEASURE SHORT FORM (PAM-SF)** 11](#_Toc129190562)

[**SECTION V. PATIENT-REPORTED OUTCOMES MEASUREMENT INFORMATION SYSTEM (PROMIS)** 13](#_Toc129190563)

[**SECTION VI. FUNCTIONAL ASSESSMENT OF CANCER THERAPY-GENERAL (FACT-G)** 15](#_Toc129190564)

[**SECTION VII. SELF-REPORTED ADHERENCE (ASK-12)** 17](#_Toc129190565)

# **SECTION I. DEMOGRAPHIC SURVEY**

***This survey is used for all participants who involve in the SAMSON pilot RCT.***

Participant ID number: ___________________________

Today’s date (DD/MM/YYYY): _______/________/__________

Your smart phone is: 🞎 iPhone, version__________________

🞎 Android, version_________________

This survey includes 18 questions and would take 5-10 minutes to complete. In this section of the questionnaire, we are interested in some background information about you. This information is used solely for the purpose of ensuring that people with a range of backgrounds are involved in the study. Please either write the answer or circle the option that best describes you or your current situation.

| 1. How old are you? __________ 2. What is your gender?    1. Male    2. Female    3. Non-binary    4. Prefer not to say 3. What is your current postcode?__________ 4. In which country were you born?    1. Australia    2. Other (specify):__________________ 5. What language do you mainly speak at home?    1. English    2. Other (please specify):_____________ 6. What is your current marital status?    1. Never married/Single    2. Married/De Facto    3. Separated/Divorced    4. Widowed 7. With whom are you currently living with? (Please select all that apply).    1. Alone    2. Partner    3. Parent(s)    4. Siblings    5. Child/children    6. Friends    7. Carer    8. Other (specify):   ____________________________   1. What is/was your occupation?   _________________________________   1. Which one of the following best describes your current employment situation?    1. Working full-time (*at least* 38 hours per week)    2. Working part-time (*less than* 38 hours per week)    3. Casual    4. Sick leave/leave of absence – temporary    5. Sick leave/leave of absence – permanent    6. Unemployed    7. Retired    8. Home duties    9. Studying    10. Other: ________________________ 2. What is the highest level of formal education that you have *completed*?    1. No formal schooling/incomplete schooling    2. Primary school    3. Secondary/High school    4. Vocational    5. University    6. Postgraduate    7. Other (specify):   _____________________________ | 1. What is your current annual personal income?    1. Nil    2. Less than $20,000    3. Between $20,000 and $50,000    4. Between $50,001 and $80,000    5. Between $80,001 and $110,000    6. Over $110,000    7. Prefer not to say 2. Thinking about your diagnosis of this cancer, what year were you first diagnosed?   (enter year first diagnosed):____________   1. What medications have you been previously prescribed for this cancer, before starting the current treatment (tick all that apply)?    1. None    2. Medication 1 specify:___________________    3. Medication 2 specify:___________________    4. Medication 3 specify:___________________ 2. In addition to your diagnosis this cancer, do you have any other chronic illnesses?    1. Yes (go to Question 15)    2. No (go to Question 17) 3. Thinking about your answer to Question 14, do you take any prescribed medications for this/these chronic illnesses?    1. Yes (go to Question 16)    2. No (go to Question 17) 4. Thinking about your answer to Question 14, what prescribed medications and dosages do you take?    1. Medication 1 name:___________________    2. Medication 1 dosage:__________(mg per day)    3. Medication 2 name:___________________    4. Medication 2 dosage:__________(mg per day)    5. Medication 3 name:___________________    6. Medication 3 dosage: _________(mg per day) 5. Do you regularly take any natural or over-the-counter treatments (e.g. vitamins, herbs, pain relief, etc.)?    1. Yes (go to Question 18)    2. No (finished Demographic Questionnaire) 6. Thinking about your answer to Question 17, what natural or over-the-counter treatments do you take and what dosage?    1. Medication 1 name:___________________    2. Medication 1 dosage:__________(mg per day)    3. Medication 2 name:___________________    4. Medication 2 dosage:__________(mg per day)    5. Medication 3 name:___________________    6. Medication 3 dosage: _________(mg per day) |
| --- | --- |

# **SECTION II. POST-INTERVENTION SURVEY FOR HEALTHCARE PROFESSIONALS**

***This survey is used for healthcare professionals involved in the SAMSON pilot RCT. This is adapted from the Unified Theory of Acceptance and Use of Technology (UTAUT) (Nguyen et al., 2016; Venkatesh et al., 2003; Venkatesh et al., 2012)***

Today’s date (DD/MM/YYYY): _______/________/__________

You are invited to complete this questionnaire to evaluate the SAMSON solution that you have been using. The SAMSON solution to improve medication adherence in cancer includes two components: the SAMSON mobile platform and the Motivational Interviewing Training Platform. This research project is supported by research funding from Peter MacCallum Cancer Centre, Digital Health Cooperative Research Centre and the Swinburne University of Technology.

By completing this survey, you are consenting for the research team to use the data from this questionnaire for the purposes of the research project. All of the information that you have provided will be anonymous. In any publication and/or presentation, information will be provided in such a way that you cannot be identified.

Your opinions are crucial for us to evaluate the SAMSON solution. When you have completed the questionnaire, please check through it to make sure you have not missed any questions.

This questionnaire includes 24 questions. It should take you no more than 15 minutes to complete.

For questions 1-17, please circle the option (numbers) that most accurately represents your views where:

1 = Strongly Disagree, 2 = Disagree, 3 = Neither, 4 = Agree, and 5 = Strongly Agree.

|  | Strongly Disagree | Disagree | Neither | Agree | Strongly Agree |
| --- | --- | --- | --- | --- | --- |
| 1. I find the SAMSON solution useful in my job | 1 | 2 | 3 | 4 | 5 |
| 1. Using the SAMSON solution enables my two-way therapeutic communication with patients | 1 | 2 | 3 | 4 | 5 |
| 1. If I use the SAMSON solution, I will increase my chance of supporting patient treatment adherence | 1 | 2 | 3 | 4 | 5 |
| 1. Overall, I find the SAMSON solution easy to use | 1 | 2 | 3 | 4 | 5 |
| 1. I find the presentation and content of the SAMSON mobile platform clear and understandable | 1 | 2 | 3 | 4 | 5 |
| 1. I can easily navigate the content on the SAMSON mobile platform | 1 | 2 | 3 | 4 | 5 |
| 1. I can easily apply motivational interviewing (MI) skills when delivering intervention consultations | 1 | 2 | 3 | 4 | 5 |
| 1. People who influence my clinical practice (my managers and senior colleagues) would be in favour of my use of the SAMSON solution | 1 | 2 | 3 | 4 | 5 |
| 1. Some of my colleagues (oncologists, nurses, pharmacists) would find the SAMSON solution valuable to use | 1 | 2 | 3 | 4 | 5 |
| 1. In general, the hospital should support the use of the SAMSON solution | 1 | 2 | 3 | 4 | 5 |
| 1. I have the resources necessary to use the SAMSON solution. | 1 | 2 | 3 | 4 | 5 |
| 1. I have the knowledge necessary to use the SAMSON solution. | 1 | 2 | 3 | 4 | 5 |
| 1. I have the knowledge necessary to use the MI technique |  |  |  |  |  |
| 1. I can get help from others when I have difficulties using the SAMSON solution | 1 | 2 | 3 | 4 | 5 |
| 1. I feel confident in using the SAMSON solution | 1 | 2 | 3 | 4 | 5 |
| 1. After this trial, I will be more likely to use the SAMSON solution if it is available in my clinical practice | 1 | 2 | 3 | 4 | 5 |
| 1. I will introduce the SAMSON solution to my colleagues | 1 | 2 | 3 | 4 | 5 |

1. What do you think about the number of consultations delivered in the SAMSON solution?

_________________________________________________________________________________________________________________________________________________________

1. What do you think about the length of consultations delivered in the SAMSON solution?

_________________________________________________________________________________________________________________________________________________________

1. What did you LIKE about the SAMSON solution? Why?

_________________________________________________________________________________________________________________________________________________________

1. What did you DISLIKE about the SAMSON solution? Why?

___________________________________________________________________________________________________________________________________________________

1. In your opinion, what we could do to make the SAMSON solution better?

____________________________________________________________________________________

____________________________________________________________________________________

1. What are the challenges that the SAMSON solution may have if it is being implemented in the clinical setting?

____________________________________________________________________________________

____________________________________________________________________________________

1. Do you have any more feedback?

____________________________________________________________________________________

____________________________________________________________________________________

# **SECTION III. POST-INTERVENTION SURVEY FOR PATIENTS**

***This survey is used for patients involved in the SAMSON pilot RCT and enrolled in the study intervention arm. This is adapted from the Unified Theory of Acceptance and Use of Technology (UTAUT) (Nguyen et al., 2016; Venkatesh et al., 2003; Venkatesh et al., 2012)***

Participant ID number: ___________________________

Today’s date (DD/MM/YYYY): _______/________/__________

You are invited to complete this questionnaire to evaluate the SAMSON solution that you have been using. The SAMSON solution to improve medication adherence in cancer includes two components: the SAMSON mobile platform and the Motivational Interviewing (MI) consultations. This research project is supported by research funding from Peter MacCallum Cancer Centre, Digital Health Cooperative Research Centre and the Swinburne University of Technology.

Your opinions are crucial for us to evaluate the SAMSON solution. When you have completed the questionnaire, please check through it to make sure you have not missed any questions.

This questionnaire includes 24 questions. It should take you no more than 15 minutes to complete.

For questions 1-18, please circle the option (numbers) that most accurately represents your views where:

1 = Strongly Disagree, 2 = Disagree, 3 = Neither, 4 = Agree, and 5 = Strongly Agree.

|  | Strongly Disagree | Disagree | Neither | Agree | Strongly Agree |
| --- | --- | --- | --- | --- | --- |
| 1. I find the SAMSON solution useful. | 1 | 2 | 3 | 4 | 5 |
| 1. The SAMSON mobile app reminds me to take medication on time | 1 | 2 | 3 | 4 | 5 |
| 1. The MI consultations could improve my knowledge of my medical condition and the treatment | 1 | 2 | 3 | 4 | 5 |
| 1. The MI consultations could increase my skills and confidence in treatment self-management | 1 | 2 | 3 | 4 | 5 |
| 1. The self-care advice in the SAMSON mobile app could increase my skills in symptom self-management | 1 | 2 | 3 | 4 | 5 |
| 1. Overall, I find the SAMSON mobile app easy to use. | 1 | 2 | 3 | 4 | 5 |
| 1. I find the content of the SAMSON mobile app clear and understandable. | 1 | 2 | 3 | 4 | 5 |
| 1. I can easily navigate the content on the SAMSON mobile app. | 1 | 2 | 3 | 4 | 5 |
| 1. My family and friends would be in favour of my use of the SAMSON solution. | 1 | 2 | 3 | 4 | 5 |
| 1. People taking oral cancer treatment like me would find the SAMSON solution valuable to use. | 1 | 2 | 3 | 4 | 5 |
| 1. In general, the hospital should support the use of the SAMSON solution for patients. | 1 | 2 | 3 | 4 | 5 |
| 1. I have the resources necessary to use the SAMSON mobile app. | 1 | 2 | 3 | 4 | 5 |
| 1. I have the knowledge necessary to use the SAMSON mobile app. | 1 | 2 | 3 | 4 | 5 |
| 1. I can get help from others when I have difficulties using the SAMSON mobile app. | 1 | 2 | 3 | 4 | 5 |
| 1. I feel confident in using the SAMSON mobile app | 1 | 2 | 3 | 4 | 5 |
| 1. After this trial, I will be more likely to use the SAMSON mobile app if it is available in my hospital | 1 | 2 | 3 | 4 | 5 |
| 1. I will introduce the SAMSON solution to people taking oral medications that I know | 1 | 2 | 3 | 4 | 5 |
| 1. I will only use the SAMSON solution if it is free | 1 | 2 | 3 | 4 | 5 |

1. What do you think about the number of MI consultations delivered in this study?

_________________________________________________________________________________________________________________________________________________________

1. What do you think about the length of MI consultations delivered in this study?

_________________________________________________________________________________________________________________________________________________________

1. What did you like about the SAMSON solution? Why?

_________________________________________________________________________________________________________________________________________________________

1. What did you dislike about the SAMSON solution? Why?

___________________________________________________________________________________________________________________________________________________

1. In your opinion, what we could do to make the SAMSON solution better?

____________________________________________________________________________________

____________________________________________________________________________________

1. Do you have any more feedback?

____________________________________________________________________________________

____________________________________________________________________________________

# **SECTION IV. PATIENT ACTIVATION MEASURE SHORT FORM (PAM-SF)**

***This survey is used for all patients involved in the SAMSON pilot RCT. This was adapted from Hibbard et al. (2005)***

Participant ID number: ___________________________

Today’s date (DD/MM/YYYY): _______/________/__________

You are invited to complete this questionnaire as part of the trial to evaluate the SAMSON intervention solution that you have received. The SAMSON solution to improve medication adherence in cancer includes the SAMSON mobile platform and the Motivational Interviewing Training Platform. This research project is supported by research funding from Peter MacCallum Cancer Centre, Digital Health Cooperative Research Centre, and Swinburne University of Technology.

Your opinions are crucial to us. When you have completed the questionnaire, please check through it to make sure you have not missed any questions.

This questionnaire includes 13 questions. It should take you approximately 5 minutes to complete. Please move the sliding scale to the point that most accurately represents your opinion.

|  | 100  1 |
| --- | --- |
| 1. When all is said and done, I am the person who is responsible for managing my health condition |  |
| 1. Taking an active role in my own health care is the most important factor in determining my health and ability to function |  |
| 1. I am confident that I can take actions that will help prevent or minimize some symptoms or problems associated with my health condition |  |
| 1. I know what each of my prescribed medications do |  |
| 1. I am confident that I can tell when I need to go get medical care and when I can handle a health problem myself |  |
| 1. I am confident I can tell my health care provider concerns I have even when he or she does not ask |  |
| 1. I am confident that I can follow through on medical treatments I need to do at home |  |
| 1. I understand the nature and causes of my health condition(s) |  |
| 1. I know the different medical treatment options available for my health condition |  |
| 1. I have been able to maintain the lifestyle changes for my health that I have made |  |
| 1. I know how to prevent further problems with my health condition |  |
| 1. I am confident I can figure out solutions when new situations or problems arise with my health condition |  |
| 1. I am confident that I can maintain lifestyle changes like diet and exercise even during times of stress |  |

# **SECTION V. PATIENT-REPORTED OUTCOMES MEASUREMENT INFORMATION SYSTEM (PROMIS)**

***This survey is used for all patients involved in the SAMSON pilot RCT. It is adapted from Northwestern University (2023) and Quach et. al. (2016)***

Participant ID number: ___________________________

Today’s date (DD/MM/YYYY): _______/________/__________

You are invited to complete this questionnaire as part of the trial to evaluate the SAMSON intervention solution that you have received. The SAMSON solution to improve medication adherence in cancer includes the SAMSON mobile platform and the Motivational Interviewing Training Platform. This research project is supported by research funding from Peter MacCallum Cancer Centre, Digital Health Cooperative Research Centre, and Swinburne University of Technology.

Your opinions are crucial to us. When you have completed the questionnaire, please check through it to make sure you have not missed any questions.

This questionnaire includes 26 questions. It should take you approximately 5 minutes to complete. Please respond to each question or statement by circling the most appropriate response per row.

| **In the past 7 days…** | **Never** | **Rarely** | **Sometimes** | **Often** | **Always** |
| --- | --- | --- | --- | --- | --- |
| I felt fearful | 1 | 2 | 3 | 4 | 5 |
| I found it hard to focus on anything other than my anxiety | 1 | 2 | 3 | 4 | 5 |
| My worries overwhelmed me | 1 | 2 | 3 | 4 | 5 |
| I felt uneasy | 1 | 2 | 3 | 4 | 5 |

| **In the past 7 days…** | **Never** | **Rarely** | **Sometimes** | **Often** | **Always** |
| --- | --- | --- | --- | --- | --- |
| I felt worthless | 1 | 2 | 3 | 4 | 5 |
| I felt helpless | 1 | 2 | 3 | 4 | 5 |
| I felt depressed | 1 | 2 | 3 | 4 | 5 |
| I felt hopeless | 1 | 2 | 3 | 4 | 5 |

| **During the past 7 days…** | **Not at all** | **A little bit** | **Somewhat** | **Quite a bit** | **Very much** |
| --- | --- | --- | --- | --- | --- |
| I feel fatigued | 1 | 2 | 3 | 4 | 5 |
| I have trouble starting things because I am tired | 1 | 2 | 3 | 4 | 5 |
| **In the past 7 days…** | **Not at all** | **A little bit** | **Somewhat** | **Quite a bit** | **Very much** |
| How run-down did you feel on average? | 1 | 2 | 3 | 4 | 5 |
| How fatigued were you on average? | 1 | 2 | 3 | 4 | 5 |

| **In the past 7 days…** | **Not at all** | **A little bit** | **Somewhat** | **Quite a bit** | **Very much** |
| --- | --- | --- | --- | --- | --- |
| How much did pain interfere with your day to day activities? | 1 | 2 | 3 | 4 | 5 |
| How much did pain interfere with work around the home? | 1 | 2 | 3 | 4 | 5 |
| How much did pain interfere with your ability to participate in social activities? | 1 | 2 | 3 | 4 | 5 |
| How much did pain interfere with your household chores? | 1 | 2 | 3 | 4 | 5 |

|  | **Without any difficulty** | **With a little difficulty** | **With some difficulty** | **With much difficulty** | **Unable to do** |
| --- | --- | --- | --- | --- | --- |
| Are you able to do chores such as vacuuming or yard work? | 5 | 4 | 3 | 2 | 1 |
| Are you able to go up and down stairs at a normal pace? | 5 | 4 | 3 | 2 | 1 |
| Are you able to go for a walk of at least 15 minutes? | 5 | 4 | 3 | 2 | 1 |
| Are you able to run errands and shop? | 5 | 4 | 3 | 2 | 1 |
|  | **Not at all** | **Very little** | **Somewhat** | **Quite a lot** | **Cannot do** |
| Does your health now limit you in doing two hours of physical labor? | 5 | 4 | 3 | 2 | 1 |
| Does your health now limit you in doing moderate work around the house like vacuuming, sweeping floors or carrying in groceries? | 5 | 4 | 3 | 2 | 1 |

| **In the past 7 days…** | **Very poor** | **Poor** | **Fair** | **Good** | **Very good** |
| --- | --- | --- | --- | --- | --- |
| My sleep quality was… | 5 | 4 | 3 | 2 | 1 |
| **In the past 7 days…** | **Not at all** | **A little bit** | **Somewhat** | **Quite a bit** | **Very much** |
| My sleep was refreshing | 5 | 4 | 3 | 2 | 1 |
| I had a problem with my sleep | 1 | 2 | 3 | 4 | 5 |
| I had difficulty falling asleep | 1 | 2 | 3 | 4 | 5 |

# **SECTION VI. FUNCTIONAL ASSESSMENT OF CANCER THERAPY-GENERAL (FACT-G)**

***This survey is used for all patients involved in the SAMSON pilot RCT. It is adapted from Cella et. al (1993)***

Participant ID number: ___________________________

Today’s date (DD/MM/YYYY): _______/________/__________

You are invited to complete this questionnaire as part of the trial to evaluate the SAMSON intervention solution that you have received. The SAMSON solution to improve medication adherence in cancer includes the SAMSON mobile platform and the Motivational Interviewing Training Platform. This research project is supported by research funding from Peter MacCallum Cancer Centre, Digital Health Cooperative Research Centre, and Swinburne University of Technology.

Your opinions are crucial to us. When you have completed the questionnaire, please check through it to make sure you have not missed any questions.

This questionnaire includes 27 questions. It should take you approximately 5-10 minutes to complete. Below is a list of statements that other people with your illness have said are important. Please circle or mark one number per line to indicate your response as it applies to the past 7 days.

| **PHYSICAL WELL-BEING** | **Not at all** | **A little bit** | **Somewhat** | **Quite a bit** | **Very much** |
| --- | --- | --- | --- | --- | --- |
| I have a lack of energy | 0 | 1 | 2 | 3 | 4 |
| I have nausea | 0 | 1 | 2 | 3 | 4 |
| Because of my physical condition, I have trouble meeting the needs of my family | 0 | 1 | 2 | 3 | 4 |
| I have pain | 0 | 1 | 2 | 3 | 4 |
| I am bothered by side effects of treatment | 0 | 1 | 2 | 3 | 4 |
| I feel ill | 0 | 1 | 2 | 3 | 4 |
| I am forced to spend time in bed | 0 | 1 | 2 | 3 | 4 |
| **SOCIAL/FAMILY WELL-BEING** | **Not at all** | **A little bit** | **Somewhat** | **Quite a bit** | **Very much** |
| I feel close to my friends | 0 | 1 | 2 | 3 | 4 |
| I get emotional support from my family | 0 | 1 | 2 | 3 | 4 |
| I get support from my friends | 0 | 1 | 2 | 3 | 4 |
| My family has accepted my illness | 0 | 1 | 2 | 3 | 4 |
| I am satisfied with family communication about my illness | 0 | 1 | 2 | 3 | 4 |
| I feel close to my partner (or the person who is my main support) | 0 | 1 | 2 | 3 | 4 |
| *Regardless of your current level of sexual activity, please answer the following question. If you prefer not to answer it, please mark this box □ and go to the next section.* | | | | | |
| I am satisfied with my sex life | 0 | 1 | 2 | 3 | 4 |
| **EMOTIONAL WELL-BEING** | **Not at all** | **A little bit** | **Somewhat** | **Quite a bit** | **Very much** |
| I feel sad | 0 | 1 | 2 | 3 | 4 |
| I am satisfied with how I am coping with my illness | 0 | 1 | 2 | 3 | 4 |
| I am losing hope in the fight against my illness | 0 | 1 | 2 | 3 | 4 |
| I feel nervous | 0 | 1 | 2 | 3 | 4 |
| I worry about dying | 0 | 1 | 2 | 3 | 4 |
| I worry that my condition will get worse | 0 | 1 | 2 | 3 | 4 |
| **FUNCTIONAL WELL-BEING** | **Not at all** | **A little bit** | **Somewhat** | **Quite a bit** | **Very much** |
| I am able to work (include work at home) | 0 | 1 | 2 | 3 | 4 |
| My work (include work at home) is fulfilling | 0 | 1 | 2 | 3 | 4 |
| I am able to enjoy life | 0 | 1 | 2 | 3 | 4 |
| I have accepted my illness | 0 | 1 | 2 | 3 | 4 |
| I am sleeping well | 0 | 1 | 2 | 3 | 4 |
| I am enjoying the things I usually do for fun | 0 | 1 | 2 | 3 | 4 |
| I am content with the quality of my life right now | 0 | 1 | 2 | 3 | 4 |

# **SECTION VII. SELF-REPORTED ADHERENCE (ASK-12)**

***This survey is used for all participants involved in the SAMSON pilot RCT. It was adapted from Matza et al (2009)***

Participant ID number: ___________________________

Today’s date (DD/MM/YYYY): _______/________/__________

You are invited to complete this questionnaire as part of the trial to evaluate the SAMSON intervention solution that you have received. The SAMSON solution to improve medication adherence in cancer includes the SAMSON mobile platform and the Motivational Interviewing Training Platform. This research project is supported by research funding from Peter MacCallum Cancer Centre, Digital Health Cooperative Research Centre, and Swinburne University of Technology.

Your opinions are crucial to us. When you have completed the questionnaire, please check through it to make sure you have not missed any questions.

This questionnaire includes 12 questions. It should take you approximately 2-3 minutes to complete. Please select the option that most accurately represents your opinion.

| **INCONVENIENCE/FORGETFULNESS** | | | | | |
| --- | --- | --- | --- | --- | --- |
| *Lifestyles* | Strongly Agree | Agree | Neutral | Disagree | Strongly Disagree |
| I just forget to take my medicines some of the time |  |  |  |  |  |
| I run out of my medicine because I don’t get refills on time. |  |  |  |  |  |
| Taking medicines more than once a day is inconvenient. |  |  |  |  |  |
| **TREATMENT BELIEFS** | | | | | |
| *Attitudes and beliefs* | Strongly Agree | Agree | Neutral | Disagree | Strongly Disagree |
| I feel confident that each one of my medicines will help me |  |  |  |  |  |
| I know if I am reaching my health goals |  |  |  |  |  |
| *Help from others* |  |  |  |  |  |
| I have someone I can call with questions about my medicines. |  |  |  |  |  |
| *Talking with healthcare team* |  |  |  |  |  |
| My doctor/nurse and I work together to make decisions |  |  |  |  |  |
| **BEHAVIOUR** | | | | | |
| Taking medicines | Strongly Agree | Agree | Neutral | Disagree | Strongly Disagree |
| Have you… |  |  |  |  |  |
| Taken a medicine more or less often than prescribed? |  |  |  |  |  |
| Skipped or stopped taking a medicine because you didn’t think it was working? |  |  |  |  |  |
| Skipped or stopped taking a medicine because it made you feel bad? |  |  |  |  |  |
| Skipped, stopped, not refilled, or taken less medicine because of the cost? |  |  |  |  |  |
| Not had medicine with you when it was time to take it? |  |  |  |  |  |
