# Supplementary material for: Digital Solution to Support Medication Adherence and Self-Management in Patients with Cancer (SAMSON): Pilot Randomized Controlled Trial
Source: JMIR Form Res. 2025 Feb 19;9:e65302. doi: 10.2196/65302 (PMC11888109; doi:10.2196/65302)
Supplement: Multimedia Appendix 4 [file formative_v9i1e65302_app4.docx]

**Table S1.**

| **Measures of feasibility** | **% Lower threshold** | **% Upper threshold** |
| --- | --- | --- |
| Recruitment rate | 30 | 60 |
| Randomisation rate | 70 | 80 |
| Retention rate | 70 | 80 |
| Intervention adherence – patients (responding to medication reminders and side-effects surveys) | 70 | 90 |
| Survey data collection compliance | 50 | 70 |
