# Supplementary material for: Digital Solution to Support Medication Adherence and Self-Management in Patients with Cancer (SAMSON): Pilot Randomized Controlled Trial
Source: JMIR Form Res. 2025 Feb 19;9:e65302. doi: 10.2196/65302 (PMC11888109; doi:10.2196/65302)
Supplement: Multimedia Appendix 5 [file formative_v9i1e65302_app5.pdf]

Peter MacCallum Cancer Centre
305 Grattan Street
Melbourne Victoria
3000 Australia

Postal Address
Locked Bag 1 A'Beckett Street
Victoria 8006 Australia

Phone +61 3 8559 5000
Fax +61 3 03 8559 7379
ABN 42 100 504 883

Locations
Melbourne
Bendigo
Box Hill
Moorabbin
Sunshine

Peter Mac
Peter MacCallum Cancer Centre
Victoria Australia

PETER MACCALLUM CANCER CENTRE HUMAN RESEARCH ETHICS COMMITTEE [EC00235]
ETHICAL APPROVAL

Peter Mac No: 23/31
HREC Reference: HREC/95332/PMCC
HREC Approval Date: 6 June 2023

Title: Pilot randomized controlled trial (RCT) to test the acceptability, feasibility and potential efficacy of the SAMSON (Safety and Adherence to Medications and Self-care advice in Oncology) intervention solution

Principal Investigator: Prof Penelope Schofield

I am pleased to advise that the above project has **received ethical approval** from the Peter MacCallum Cancer Centre Human Research Ethics Committee (HREC). The HREC confirms that your proposal meets the requirements of the National Statement on Ethical Conduct in Human Research (2018). This HREC is organised and operates in accordance with the National Health and Medical Research Council's (NHRMC) National Statement on Ethical Conduct in Human Research (2018), and all subsequent updates, and in accordance with the Note for Guidance on Good Clinical Practice (CPMP/ICH/135/95), the Health Privacy Principles described in the Health Records Act 2001 (Vic) and Section 95A of the Privacy Act 1988 (and subsequent Guidelines).

Ethical approval for this project applies at the following sites:

Site
Peter MacCallum Cancer Centre

Approved Documents
The following documents have been reviewed and approved:

Document	Version	Date
Protocol *SAMSON*	1.0	2 May 2023
Participant Information Sheet and Consent Form	1.0	2 May 2023
Survey Booklet	1.0	2 May 2023
Mobile App Participant User Manual	1.0	8 March 2023
HP Website User Manual	1.0	8 March 2023
Pharmacist Intervention Manual	1.0	8 March 2023
Nurse Intervention Manual	1.0	8 March 2023
Participant Spreadsheet	--	Undated
Recruitment Flyer	1.0	2 May 2023

Governance Authorisation

Governance Authorisation is required at each site participating in the study before the research project can commence at that site. You are required to provide a copy of this HREC approval letter to the principal investigator for each site covered by this ethics approval for inclusion in the site specific assessment application.

Conditions of Ethical Approval

- You are required to submit to the HREC:
  - An Annual Progress Report (that covers all sites listed on the approval) for the duration of the project. This report is due on the anniversary of HREC approval. Continuation of ethics approval is contingent on submission of an annual report, due within one month of the approval anniversary. Failure to comply with this requirement may result in suspension of the project by the HREC.
  - A comprehensive Final Report upon completion of the project.
- Submit to the reviewing HREC for approval any proposed amendments to the project including any proposed changes to the Protocol, Participant Information and Consent Form/s and the Investigator Brochure.
- Notify the reviewing HREC of any adverse events that have a material impact on the conduct of the research in accordance with the NHMRC Position Statement: *Safety monitoring and reporting in clinical trials involving therapeutic products November 2016*.
- Notify the reviewing HREC of your inability to continue as Coordinating Principal Investigator.
- Notify the reviewing HREC of the failure to commence the study within 12 months of the HREC approval date or if a decision is taken to end the study at any of the sites prior to the expected date of completion.
- Notify the reviewing HREC of any matters which may impact the conduct of the project.
- If your project involves radiation, you are legally obliged to conduct your research in accordance with the Australian Radiation Protection and Nuclear Safety Agency Code of Practice 'Exposure of Humans to Ionizing Radiation for Research Purposes' Radiation Protection series Publication No.8 (May 2005)(ARPANSA Code).

Please note: Template forms for reporting Amendments, Adverse events, Annual/Final reports, etc. can be accessed from: www.petermac.org/research/doing-research-us/ethics-governance.

The HREC may conduct an audit of the project at any time.

Yours sincerely,

Dianne Snowden

Digitally signed by Dianne Snowden
Date: 2023.06.06 12:31:50 +10'00'

Dr Dianne Snowden
Manager, Human Research Ethics & Governance
T: 8559 7540
E: ethics@petermac.org
