# Supplementary material for: Digital Solution to Support Medication Adherence and Self-Management in Patients with Cancer (SAMSON): Pilot Randomized Controlled Trial
Source: JMIR Form Res. 2025 Feb 19;9:e65302. doi: 10.2196/65302 (PMC11888109; doi:10.2196/65302)
Supplement: Multimedia Appendix 6 [file formative_v9i1e65302_app6.pdf]

Swinburne University Human Research Ethics Committee

Approval Certificate

19/06/2023

Dear Penelope,

Ref: 20237273-15836 :SAMSON pilot randomized controlled trial

Approved Duration: 19/06/2023 to 30/06/2024

External HREC reference: Peter MacCallum Cancer Centre ref: HREC/95332/PMCC

I refer to the application submitted for Swinburne ethics clearance for the above project.

Relevant submissions pertaining to the applications was given expedited ethical review on behalf of Swinburne University's Human Research Ethics Committee (SUHREC) by a committee delegate, significantly on the basis of the ethical review conducted by the Peter MacCallum Cancer Centre HREC/95332/PMCC.

I am pleased to advise that, as submitted to date and as regards Swinburne, ethics clearance has been given for the above project to proceed in line with standard on-going ethics clearance conditions outlined below and as follows. The Peter MacCallum Cancer Centre HREC may need to be advised of this approval.

- The approved duration is as stated above unless an extension request is subsequently approved.
- All human research activity undertaken under Swinburne auspices must conform to Swinburne and external regulatory standards, including the *National Statement on Ethical Conduct in Human Research* and with respect to secure data use, retention and disposal.
- The named Swinburne Chief Investigator/Supervisor remains responsible for any personnel appointed to or associated with the project being made aware of ethics clearance conditions, including research and consent procedures or instruments approved. Any change in Chief Investigator/Supervisor, and addition or removal of other personnel/students from the project, requires timely notification and SUHREC endorsement.
- The above project has been approved as submitted for ethical review by or on behalf of SUHREC. Amendments to approved procedures or instruments ordinarily require prior ethical appraisal/clearance from the Peter MacCallum Cancer Centre HREC HREC before being submitted to SUHREC for approval. SUHREC must be notified immediately or as soon as possible thereafter of (a) any serious or unexpected adverse effects on participants and any redress measures; (b) proposed changes in protocols; and (c) unforeseen events which might affect continued ethical acceptability of the project.
- At a minimum, an annual report on the progress of the project is required as well as at the conclusion (or abandonment) of the project. However, formats required by or submissions to the Peter MacCallum Cancer Centre HREC in this regard may be acceptable.
- A duly authorised external or internal audit of the project may be undertaken at any time.

Chief Investigator

Penelope Schofield

Student Investigators

Thu Ha Dang

Please contact the Swinburne Research Ethics Office if you have any queries.

Regards,

Dr Astrid Nordmann

on behalf of the Swinburne University Human Research Ethics Committee

Research Ethics Office

Swinburne University of Technology

P: +61 3 9214 3845 | E: resethics@swin.edu.au
