# Supplementary material for: Digital Solution to Support Medication Adherence and Self-Management in Patients with Cancer (SAMSON): Pilot Randomized Controlled Trial
Source: JMIR Form Res. 2025 Feb 19;9:e65302. doi: 10.2196/65302 (PMC11888109; doi:10.2196/65302)
Supplement: Multimedia Appendix 7 [file formative_v9i1e65302_app7.doc]

**Table S2.**

| **Characteristics** | **Total**  **(N=31)** | **Intervention (N=15)** | **Control (N=16)** |
| --- | --- | --- | --- |
| **Age (years)** |  |  |  |
| Mean (SD) | 58.1 (13.7) | 59.1 (13.1)^*^ | 57.2 (14.6)^*^ |
| **Sex, n (%)** |  |  |  |
| Male | 21 (68) | 9 (60)^*^ | 12 (75)^*^ |
| **Country of birth, n (%)** |  |  |  |
| Australia/New Zealand | 21 (68) | 11 (74) | 10 (63) |
| America | 2 (7) | 1 (7) | 1 (6) |
| Asia | 4 (13) | 3 (20) | 1 (6) |
| Europe | 4 (13) |  | 4 (25) |
| **English as first language, n (%)** |  |  |  |
| Yes | 28 (90) | 13 (87)^*^ | 15 (94)^*^ |
| No | 3 (10) | 2 (13)^*^ | 1 (6)^*^ |
| **Education (highest level completed), n (%)** |  |  |  |
| Secondary/high school | 8 (26) | 3 (20)^*^ | 5 (31)^*^ |
| Vocational | 2 (7) | 1 (7)^*^ | 1 (6)^*^ |
| University | 11 (36) | 6 (40)^*^ | 5 (31)^*^ |
| Postgraduate diploma/masters/PhD | 10 (32) | 5 (33)^*^ | 5 (31)^*^ |
| **Income (AU$), n (%)** |  |  |  |
| Nil | 2 (7) | 1 (7)^*^ | 1 (7)^*^ |
| ≤20,000 | 4 (13) | 1 (7)^*^ | 3 (20)^*^ |
| 20,001-50,000 | 4 (13) | 3 (20)^*^ | 1 (7)^*^ |
| 50,001-80,000 | 5 (16) | 3 (20)^*^ | 2 (13)^*^ |
| 80,001-110,000 | 1 (3) | 1 (7) |  |
| >110,000 | 6 (19) | 2 (13)^*^ | 4 (27)^*^ |
| Prefer not to say | 9 (29) | 4 (27)^*^ | 5 (31)^*^ |
| **Residence** |  |  |  |
| Metropolitan of Victoria state | 25 (81) | 11 (73)^*^ | 14 (88)^*^ |
| Rural of Victoria state | 5 (16) | 4 (27)^*^ | 1 (6)^*^ |
| Interstate | 1 (3) |  | 1 (6) |
| **Time since diagnosis** |  |  |  |
| Median (Range) | 4.0 (1-22) | 6.9 (1-22)^*^ | 5.9 (1-20)^*^ |
| **Diagnosis, n (%)** |  |  |  |
| Acute leukemia (AML/ALL) | 3 (10) | 2 (13) | 1 (6) |
| Chronic lymphocytic leukemia (CLL/SLL) | 8 (26) | 4 (27) | 4 (25) |
| Chronic myeloid leukemia (CML) | 5 (16) | 3 (20) | 2 (13) |
| Lymphoma non-Hodgkin | 7 (23) | 1 (7) | 6 (38) |
| Multiple myeloma | 4 (13) | 1 (7) | 3 (19) |
| Myeloproliferative neoplasm (MPN) | 3 (10) | 3 (20) |  |
| Myelodysplastic syndrome (MDS) | 1 (3) | 1 (7) |  |
| **Comorbidity, n (%)** |  |  |  |
| Having chronic condition(s) other than  cancer | 15 (48) | 8 (53)^*^ | 7 (44)^*^ |
| Taking oral medication(s) for the chronic  condition(s) other than cancer | 11 (36) | 8 (53) | 3 (19) |
| **Phone operating system, n (%)** |  |  |  |
| Android | 13 (42) | 4 (27) | 9 (56) |
| iOS | 18 (58) | 11 (73) | 7 (44) |
| **People living with the participant, n (%)** |  |  |  |
| Nil | 4 (13) | 2 (13) | 2 (13) |
| Partner | 25 (81) | 12 (80) | 13 (81) |
| Friend | 1 (3) | 1 (7) |  |
| Not provided | 1 (3) |  | 1 (6) |

*^*^ There were no significant differences between attributes at p<0.05*
