# Supplementary material for: Digital Solution to Support Medication Adherence and Self-Management in Patients with Cancer (SAMSON): Pilot Randomized Controlled Trial
Source: JMIR Form Res. 2025 Feb 19;9:e65302. doi: 10.2196/65302 (PMC11888109; doi:10.2196/65302)
Supplement: Multimedia Appendix 8 [file formative_v9i1e65302_app8.docx]

Supplementary 8. Patient acceptance and use of the SAMSON solution using adapted Unified Theory of Acceptance and Use of Technology (UTAUT) [1]

| Constructs and questions | Strongly disagree  n (%) | Disagree  n (%) | Neither  n (%) | Agree  n (%) | Strongly agree  n (%) |
| --- | --- | --- | --- | --- | --- |
| Performance expectancy | | | | | |
| 1. I find the SAMSON solution useful | 1 (8.3) | 2 (16.7) | 3 (25.0) | 4 (33.3) | 2 (16.7) |
| 1. The SAMSON mobile app reminds me to take medication on time |  | 3 (25.0) | 2 (16.7) | 4 (33.3) | 3 (25.0) |
| 1. The MI consultations could improve my knowledge of my medical condition and the treatment |  |  | 2 (16.7) | 8 (66.7) | 2 (16.7) |
| 1. The MI consultations could increase my skills and confidence in treatment self-management |  |  | 6 (50.0) | 5 (41.7) | 1 (8.3) |
| 1. The self-care advice in the SAMSON mobile app could increase my skills in symptom self-management |  |  | 3 (25.0) | 8 (66.7) | 1 (8.3) |
| Effort expectancy | | | | | |
| 1. Overall, I find the SAMSON mobile app easy to use |  | 1 (8.3) | 2 (16.7) | 5 (41.7) | 4 (33.3) |
| 1. I find the content of the SAMSON mobile app clear and understandable |  |  | 1 (8.3) | 8 (66.7) | 3 (25.0) |
| 1. I can easily navigate the content on the SAMSON mobile app |  | 1 (8.3) | 1 (8.3) | 7 (58.3) | 3 (25.0) |
| Social influence | | | | | |
| 1. My family and friends would be in favour of my use of the SAMSON solution |  |  | 7 (58.3) | 4 (33.3) | 1 (8.3) |
| 1. People taking oral cancer treatment like me would find the SAMSON solution valuable to use |  | 1 (8.3) | 1 (8.3) | 8 (66.7) | 2 (16.7) |
| 1. In general, the hospital should support the use of the SAMSON solution for patients |  |  | 1 (8.3) | 10 (83.3) | 1 (8.3) |
| Facilitating conditions | | | | | |
| 1. I have the resources necessary to use the SAMSON mobile app. |  |  | 2 (16.7) | 7 (58.3) | 3 (25.0) |
| 1. I have the knowledge necessary to use the SAMSON mobile app |  | 2 (16.7) |  | 7 (58.3) | 3 (25.0) |
| 1. I can get help from others when I have difficulties using the SAMSON mobile app |  |  | 3 (25.0) | 7 (58.3) | 2 (16.7) |
| Behavioural intention | | | | | |
| 1. I feel confident in using the SAMSON mobile app |  | 1 (8.3) | 2 (16.7) | 4 (33.3) | 5 (41.7) |
| 1. After this trial, I will be more likely to use the SAMSON mobile app if it is available in my hospital | 3 (25.0) |  | 1 (8.3) | 5 (41.7) | 3 (25.0) |
| 1. I will introduce the SAMSON solution to people taking oral medications that I know | 1 (8.3) | 2 (16.7) | 2 (16.7) | 5 (41.7) | 2 (16.7) |
| 1. I will only use the SAMSON solution if it is free |  |  | 4 (33.3) | 5 (41.7) | 3 (25.0) |

Reference

1. Venkatesh V, Morris MG, Davis G, Davis F. User acceptance of information technology: Toward a unified view. MIS Quarterly. 2003;27(3):425-78.
