# Supplementary material for: Digital Solution to Support Medication Adherence and Self-Management in Patients with Cancer (SAMSON): Pilot Randomized Controlled Trial
Source: JMIR Form Res. 2025 Feb 19;9:e65302. doi: 10.2196/65302 (PMC11888109; doi:10.2196/65302)
Supplement: Multimedia Appendix 9 [file formative_v9i1e65302_app9.docx]

Supplementary 9. Healthcare professionals acceptance and use of the SAMSON solution using adapted Unified Theory of Acceptance and Use of Technology (UTAUT) [1]

| Constructs and questions | Strongly disagree  n (%) | Disagree  n (%) | Neither  n (%) | Agree  n (%) | Strongly agree  n (%) |
| --- | --- | --- | --- | --- | --- |
| Performance expectancy | | | | | |
| 1. I find the SAMSON solution useful in my job |  |  | 1 (33.3) | 1 (33.3) | 1 (33.3) |
| 1. Using the SAMSON solution enables my two-way therapeutic communication with patients |  |  |  | 2 (66.7) | 1 (33.3) |
| 1. If I use the SAMSON solution, I will increase my chance of supporting patient treatment adherence |  |  |  | 2 (66.7) | 1 (33.3) |
| Effort expectancy | | | | | |
| 1. Overall, I find the SAMSON solution easy to use |  |  |  | 2 (66.7) | 1 (33.3) |
| 1. I find the presentation and content of the SAMSON mobile platform clear and understandable |  |  | 1 (33.3) | 1 (33.3) | 1 (33.3) |
| 1. I can easily navigate the content on the SAMSON mobile platform |  |  | 2 (66.7) | 1 (33.3) |  |
| 1. I can easily apply motivational interviewing (MI) skills when delivering intervention consultations |  |  | 1 (33.3) | 1 (33.3) | 1 (33.3) |
| Social influence | | | | | |
| 1. People who influence my clinical practice (my managers and senior colleagues) would be in favour of my use of the SAMSON solution |  |  | 1 (33.3) | 1 (33.3) | 1 (33.3) |
| 1. Some of my colleagues (oncologists, nurses, pharmacists) would find the SAMSON solution valuable to use |  |  |  | 2 (66.7) | 1 (33.3) |
| 1. In general, the hospital should support the use of the SAMSON solution |  |  |  | 2 (66.7) | 1 (33.3) |
| Facilitating conditions | | | | | |
| 1. I have the resources necessary to use the SAMSON solution |  |  | 1 (33.3) | 1 (33.3) | 1 (33.3) |
| 1. I have the knowledge necessary to use the SAMSON solution |  |  | 1 (33.3) | 1 (33.3) | 1 (33.3) |
| 1. I have the knowledge necessary to use the MI technique |  |  |  | 1 (33.3) | 2 (66.7) |
| 1. I can get help from others when I have difficulties using the SAMSON solution |  |  |  | 2 (66.7) | 1 (33.3) |
| Behavioural intention | | | | | |
| 1. I feel confident in using the SAMSON solution |  |  | 1 (33.3) | 1 (33.3) | 1 (33.3) |
| 1. After this trial, I will be more likely to use the SAMSON solution if it is available in my clinical practice |  |  | 1 (33.3) | 1 (33.3) | 1 (33.3) |
| 1. I will introduce the SAMSON solution to my colleagues |  |  |  | 2 (66.7) | 1 (33.3) |

Reference

1. Venkatesh V, Morris MG, Davis G, Davis F. User acceptance of information technology: Toward a unified view. MIS Quarterly. 2003;27(3):425-78.
