# Supplementary material for: Digital Solution to Support Medication Adherence and Self-Management in Patients with Cancer (SAMSON): Pilot Randomized Controlled Trial
Source: JMIR Form Res. 2025 Feb 19;9:e65302. doi: 10.2196/65302 (PMC11888109; doi:10.2196/65302)
Supplement: Multimedia Appendix 10 [file formative_v9i1e65302_app10.docx]

**Table S3.**

| **Feasibility measurements** | **Lower threshold (%)** | **Upper threshold (%)** | **Results (%)** | **Conclusions** |
| --- | --- | --- | --- | --- |
| Recruitment rate | 30 | 60 | 79 | Feasible |
| Randomisation rate | 70 | 80 | 94 | Feasible |
| Retention rate of study | 70 | 80 | 85 | Feasible |
| Intervention adherence – patients | | | | |
| - Responding to   medication reminders | 70 | 90 | 69 | Infeasible |
| - Responding to   symptoms surveys | 70 | 90 | 91 | Feasible |
| Data collection compliance: | | | | |
| - Baseline | | | | |
| ASK-12 | 50 | 70 | 94 | Feasible |
| PAM-SF | 50 | 70 | 94 | Feasible |
| PROMIS | 50 | 70 | 94 | Feasible |
| FACT-G | 50 | 70 | 94 | Feasible |
| - 12 weeks | | | | |
| ASK-12 | 50 | 70 | 100 | Feasible |
| PAM-SF | 50 | 70 | 100 | Feasible |
| PROMIS | 50 | 70 | 100 | Feasible |
| FACT-G | 50 | 70 | 100 | Feasible |
| UTAUT | 50 | 70 | 100 | Feasible |
