# Supplementary material for: Digital Solution to Support Medication Adherence and Self-Management in Patients with Cancer (SAMSON): Pilot Randomized Controlled Trial
Source: JMIR Form Res. 2025 Feb 19;9:e65302. doi: 10.2196/65302 (PMC11888109; doi:10.2196/65302)
Supplement: Multimedia Appendix 11 [file formative_v9i1e65302_app11.docx]

Table S4.

| Secondary outcomes | Control at  Baseline  *Mean (SD)*  *n=16* | Control at  Follow-up  *Mean (SD)*  *n=16* | Intervention at Baseline  *Mean (SD)*  *n=15* | Intervention Follow-up  *Mean (SD)*  *n=12* | β (95% CI) |
| --- | --- | --- | --- | --- | --- |
| ASK-12 | 3.10 (0.49) | 3.45 (0.47) | 2.99 (0.43) | 3.37 (0.49) | -0.06 (-0.40, 0.28) |
| PAM-SF | 3.13 (0.47) | 3.19 (0.46) | 3.23 (0.31) | 3.35 (0.26) | 0.05 (-0.17, 0.27) |
| PROMIS   - Anxiety - Depression - Fatigue - Pain - Sleep - Function | 2.03 (0.66)  1.95 (0.85)  2.88 (0.99)  1.92 (1.05)  2.84 (0.81)  3.66 (1.35) | 1.94 (0.74)  1.84 (0.87)  2.69 (0.77)  1.89 (1.00)  2.59 (0.82)  3.76 (1.27) | 2.35 (0.89)  2.18 (0.89)  3.22 (1.26)  2.08 (1.26)  3.02 (1.02)  3.37 (1.32) | 1.79 (0.74)  1.67 (0.69)  2.65 (1.08)  1.71 (0.95)  2.52 (0.53)  3.94 (1.03) | -3.52 (-9.62, 2.57)  -3.27 (-8.11, 1.58)  -1.88 (-6.12, 2.36)  -1.20 (-7.82, 5.43)  -1.59 (-7.38, 4.21)  1.31 (-0.97, 3.59) |
| FACT-G   - Physical well-being - Social/family well-being - Emotional well-being - Functional well-being | 1.29 (0.91)  3.23 (0.79)  1.36 (0.55)  2.24 (0.98) | 1.25 (0.88)  3.03 (0.85)  1.31 (0.53)  2.36 (0.97) | 1.30 (0.80)  3.20 (0.77)  1.41 (0.54)  2.20 (0.76) | 0.93 (0.63)  3.19 (0.66)  1.19 (0.42)  2.54 (0.78) | -0.20 (-0.49, 0.09)  0.22 (-0.08, 0.52)  -0.05 (-0.38, 0.27)  0.13 (-0.25, 0.51) |
